# Predictive capability evaluation and mechanism of Ce (III) extraction using solvent extraction with Cyanex 572

**DOI:** 10.1038/s41598-022-14528-9

**Published:** 2022-06-20

**Authors:** Ebrahim Allahkarami, Bahram Rezai, Rama Rao Karri, Nabisab Mujawar Mubarak

**Affiliations:** 1 Department of Mining Engineering, Amirkabir University of Technology, Tehran, Iran; 2 Petroleum and Chemical Engineering, Faculty of Engineering, Universiti Teknologi Brunei, Bandar Seri Begawan, Brunei Darussalam

**Keywords:** Environmental sciences, Hydrology

## Abstract

Owing to the high toxicity of cerium toward living organisms, it is necessary to remove cerium from aqueous solutions. In this regard, the extraction of cerium (Ce (III)) from nitrate media by Cyanex 572 under different operating conditions was examined in this study. The effect of contact time, pH, extractant concentration, and nitrate ion concentration were investigated to characterize the extraction behavior of cerium and based on these outcomes, an extraction mechanism was suggested. The analysis of infrared spectra of Cyanex 572 before and after the extraction of cerium indicated that cerium extraction was performed via a cation-exchange mechanism. Then, the predictive models based on intelligent techniques [artificial neural network (ANN) and hybrid neural-genetic algorithm (GA-ANN)] were developed to predict the cerium extraction efficiency. The GA-ANN model provided better predictions that resulted higher R^2^ and lower MSE compared to ANN model for predicting the extraction efficiency of cerium by Cyanex 572. The interactive effects of each process variable on cerium extraction were also investigated systematically. pH was the most influential parameter on cerium extraction, followed by extractant concentration, nitrate ion concentration and contact time. Finally, the separation of cerium from other rare earth elements like La (III), Nd (III), Pr (III), and Y (III) was conducted and observed that the present system provides a better separation of cerium from rare heavy earth than light rare earths.

## Introduction

Cerium, one of the most abundant elements in the lanthanide series, has been widely used in many applications, such as catalysts, fluorescent powders, alloys, solar panels, and many more^1,2^. Given the physicochemical properties of cerium, the world consumption of cerium has been increasing during the last decades. Its increased demand has led to high public contact with it. Therefore, more cerium has entered the ecosystem and accumulated in the environment, and this will eventually result in increased concentration of cerium in animals, human body, aquatic creatures, and soil particles^3^. A high concentration of cerium can cause lung embolisms, necrotic changes in the liver, negative effects in reproduction, and alters nervous system activities^1^. The toxicity range of cerium compounds is found to be in the range of low to moderate. To protect public health from severe environmental problems, the Occupational Safety and Health Administration [OSHA (PEL)] recommends the optimum cerium extent of 15 mg/m^3^ in safe water^4^. To ensure the proper quality of treated effluent for various purposes, it is necessary to remove cerium from aqueous solutions effectively. There are different methods such as membrane separation^5,6^, solvent extraction^7,8^, ion exchange^9^, and adsorption^10^ that can be applied to extract cerium from an aqueous solution. Precipitation is a useful method for the separation of Ce (IV) from the aqueous solution. Furthermore, the other disadvantageous of precipitation method are sludge production, accurate control of the solution pH during the process, extra cost for sludge disposal. The advantages and disadvantages of these methods for cerium extraction/removal were discussed in the literature^1^. Among them, solvent extraction is the most versatile commercial method for separating Ce (III) because it can handle many diluent pregnant liquors^11,12^. For this purpose, different solvents such as acidic organophosphorus extractants^13,14^, neutral organophosphorus extractants^15^, high molecular weight amines^16,17^, and others^18,19^ have been used for the extraction of cerium from aqueous solutions.

For instance, the extraction behavior of Ce (III) and Sm(III) from different aqueous media (nitric acid, sulfuric acid, and hydrochloric acid) using 2-ethyl hexyl phosphonic acid mono-2-ethyl hexyl ester (PC88A) was investigated^20^. These studies indicated that HCl and HNO_3_ were suitable aqueous media for the extraction of these metals. Mishra and Sahu^21^ reported the synergistic extraction of Ce (III) from dilute nitric acid medium with a mixture of tri-octyl phosphine oxide (Cyanex 921) and PC88A. They found that the extracted species contain two molecules of PC88A and one molecule of Cyanex 921. Khodakarami and Alagha^22^ synthesized two novel functionalized ionic liquids and used them to extract rare earth elements from aqueous solutions.

Stripping studies showed that HCl was found to be favorable for both FILs. However, organophosphorus extractants have attracted great attention in recent years due to suitable loading and stripping characteristics, fast kinetics, and their chemical stability^11,12^. Cytec Industries has released a new extractant called Cyanex 572, a mixture of phosphonic and phosphinic acids^23,24^. Nie et al.^24^ studied the solvent extraction of Sc (III) from a leaching solution of tungsten residue using Cyanex 572, Cyanex 923 [a mixture of tertiary octyl and hexyl phosphine oxides, i.e., dioctyl-monohexyl phosphine oxide (40–44%), mono-octyldihexyl phosphine oxide (28–32%) and tri-n-octyl phosphine oxide (12–16%)], tri-n-butyl phosphate (TBP), and 2-ethylhexyl phosphonic acid mono-2-ethylhexl ester (P507). They applied the mentioned solvents for the extraction of Sc (III) from the leaching solution of tungsten residue ([Sc] = 9.9 mg/L, [Th] = 8.9 mg/L, [Ti] = 30.7 mg/L, [Zr] = 1.3 mg/L, [Fe] = 13,091.4 mg/L, [Mn] = 9530.9 mg/L, [Res] = 40.5 mg/L, [Al] = 506.5 mg/L, [Ca] = 5591.1, and [Mg] = 221.1 mg/L). The extraction of Sc (III) from leaching solution of tungsten residue by each extractant was examined at different extractant concentration (0.04–0.28 mol/L) and the solution acidity of 1.38 mol/L H^+^. Their results indicated that Cyanex 572 performed better than P507, Cyanex 923 and TBP because of its better extraction and stripping properties^24^.

Increasing the overall REE production to meet the highest demand of any REE and to stockpile the other REEs with lower demand, can create a balance between REE demand and the production and cost of REE. The market of REE is preferentially driven by the demand for abundant elements such as cerium and lanthanum because it will resulted in less problems with stockpiling of the elements that are available in excess^25,26^. Due to the increasing demand for cerium, applying a technique for managing this metal is of great importance. For this purpose, automatic control for the quality control of chemical processes and the optimization of energy and material by online measurements is necessary^27^. The online measurement of parameters in chemical processes requires high-cost instruments. Also, the investments require a significant amount of maintenance and calibration work. Therefore, statistical and artificial intelligence methods have been developed for monitoring purposes. These methods have been effectively applied in many processes^28,29^, especially in chemical engineering^30^. It has been proved that artificial intelligence methods outperform statistical techniques to predict process outputs^31^. The use of artificial intelligence methods such as artificial neural network (ANN)^32^, support vector machine (SVM)^33^, and adaptive neural-based fuzzy inference system (ANFIS)^34^ for modeling and simulation of chemical processes has attracted great attention in recent years. Among them, ANNs have been successfully applied in chemical processes such as flotation^28^, liquid–liquid extraction^35,36^, adsorption^32^, and so on^29,37^. Usually, the neural network training is done using random initial weights. This can result in trapping into the local minima and slow convergence speed in the training phase^38^. Therefore, this research contributes to the combination of genetic algorithms and artificial neural networks for improving the neural network's performance for the prediction of process outputs.

There is no systematic report about the cerium extraction from nitrate media by Cyanex 572^®^. The present research objectives are to extract Ce (III) from nitrate media by Cyanex 572^®^ and to develop the process for the separation of Ce (III) from other rare earths using this extraction system. The influence of operating parameters, i.e., pH, contact time, extract concentration, and nitrate ion concentration on cerium extraction, was investigated. The possible mechanism between Ce (III) and Cyanex 572 was investigated using graphical analysis and validated by Fourier Transform Infrared Spectroscopy (FTIR) analysis. After that, operating parameters were applied to develop predictive models based on artificial neural networks (ANN) and hybrid neural-genetic algorithms (GA-ANN). The developed models' accuracy was evaluated using mean square error (MSE) and coefficient of determination (R^2^). Finally, Ce (III) separation from other rare earths using this extraction system was investigated.

## Materials and methods

### Experimental procedure

Aqueous solutions containing rare earths were prepared by dissolving their corresponding nitrates (99.99%, Sigma Aldrich) in double-distilled water. Cyanex 572 extractant (chemical structure not reported) was kindly supplied by Cytec industries and used without further purification. Kerosene, from Fluka (Honeywell research chemicals), was used in the experiments as a diluent. All chemicals used were of analytical grade. A series of experiments was carried out via Cyanex 572 extractant to investigate the influence of operating parameters on the extraction of cerium. Aqueous (A) and organic (O) solutions (total volume = 10 ml and A/O = 1) were mixed at room temperature using a magnetic stirrer (300 rpm). The pH of the aqueous solution was adjusted to the desired value by dropwise addition of 0.1 M NH_4_OH and 0.1 M HNO_3_ solutions. After the separation phases using a separatory funnel, the concentrations of metals in the aqueous solutions were determined with an Agilant ICP-AES spectrometer. The organic solution was analyzed by Shimadzu FTIR spectrometer (500–3500 cm^−1^). The extraction efficiency (E%) and distribution coefficient (D) is calculated as follows:

1 $$E\% = \frac{{\left[ {Ce} \right]_{init} - \left[ {Ce} \right]_{eq} }}{{\left[ {Ce} \right]_{init} }} \times 100$$

2 $$D = \frac{{\left[ {Ce} \right]_{org} }}{{\left[ {Ce} \right]_{aq} }}$$

where [Ce]_init_ and [Ce]_eq_ represent cerium ion's initial and equilibrium concentration in the aqueous solution. Also, [Ce]_org_ and [Ce]_aq_ represent the concentration of cerium metal in the organic and aqueous phases, respectively.

To evaluate the selectivity of the extraction system, the separation factor (β) as an important parameter was used, which its equation could be expressed as^39^:

3 $$\beta_{{\frac{{REE_{A} }}{{REE_{B} }}}} = \frac{{D_{{REE_{A} }} }}{{D_{{REE_{B} }} }}$$

where D_REEA_ and D_REEB_ are the distribution coefficients of REE_A_ and REE_B_, respectively. To evaluate the separation of cerium (III) from the other REEs using Cyanex 572, a solution containing five REEs ([Ce] = 0.04 M, [Nd] = 0.015 M, [Pr] = 0.004 M, [La] = 0.014 M, and [Y] = 0.008 M) was prepared. It is noted that the mentioned concentrations are similar to the actual concentrations of REEs in the leach liquor solution obtained from an industrial process (Esfordi Mining Complex). Esfordi phosphate plant is a subsidiary of Iran Minerals Production and Supply Company located in Bafq County of Yazd Province. The Esfordi deposit includes three apatite-bearing zones, namely, the apatite-iron zone (12% P_2_O_5_), the main apatite zone (16.3% P_2_O_5_), and the tremolite-actinolite zone (14.4% P_2_O_5_). The ore reserve of the Esfordi deposit is estimated at 17 million tonnes with an average grade of 13% P_2_O_5_^40^.

### Slope analysis

The extracted Ce (III) complex with Cyanex 572 can be determined by graphical analysis^41,42^. Cyanex 572 exists as a dimer in kerosene^43^, and Ce (III) extraction with Cyanex 572 may be represented by the following equation:

4 $$Ce^{3 + } + x\left( {HL} \right)_{2\left( o \right)} \rightleftarrows Ce.H_{2x - i} L_{2x\left( o \right)} + iH_{\left( a \right)}^{ + }$$

The distribution ratio and equilibrium constant for the above reaction can be presented as follows:

5 $$K_{eq} = \frac{{\left[ {Ce.H_{2x - i} L_{2x\left( o \right)} } \right]\left[ {H^{ + } } \right]^{i} }}{{[Ce^{3 + } ] .[\left( {HL} \right)_{2\left( o \right)} ]^{x} }}$$

6 $$D = \frac{{\left[ {Ce.H_{2x - i} L_{2x\left( o \right)} } \right]}}{{[Ce^{3 + } ]}}$$

By combining Eqs. (5) and (6), the relationship between the logarithmic values of D and K_eq_ could be presented by,

7 $$\log D = x\log \left[ {\left( {HL} \right)_{2\left( o \right)} } \right] + ipH + \log K_{eq}$$

Several experiments were carried out to determine the values of x and i for obtaining the stoichiometry of the extraction reaction.

### Artificial neural network

Artificial neural networks, which are inspired by brain activity, consist of many basic processing units called neurons. These neurons are located in the layers of ANN structure (input layer, hidden layers, and output layer). These neurons are connected to all neurons in the following and preceding layers by weighted links and biases. The activation function is used to sum up, the weighted links along with bias and pass the neurons' weighted output to the hidden layer^35,44^.

In this research, the most influential variables on cerium extraction efficiency, including contact time, pH, extractant concentration, and nitrate ion concentration, were considered as the inputs to the network for predicting the extraction efficiency of cerium (output). A correlation matrix based on Pearson's correlation coefficients was produced to evaluate the strength of the linear relationships between operating variables and extraction efficiency. The obtained results are presented in Table 1. The results indicated that the extraction efficiency of cerium has a strong relationship with pH and extractant concentration. Also, there is a positive correlation coefficient between the extraction efficiency of cerium and nitrate ion concentration.

**Table 1:** Pearson’s correlation coefficients between operating parameters and extraction efficiency.

	pH	Extractant concentration	Nitrate ion concentration	Contact time	Extraction efficiency
pH	1.000	–	–	–	–
Extractant concentration	0.012	1.000	–	–	–
Nitrate ion concentration	− 0.001	0.005	1.000	–	–
Contact time	0.137	-0.031	0.025	1.000	–
Extraction efficiency	0.854	0.353	0.183	0.072	1.000

To develop a model for the prediction of cerium extraction, experimental data were used. The range of inputs and output parameters and their summary statistics are listed in Table 2.

**Table 2:** Range of studied variables and the statistics summary (number of observations was 39).

Variable		Min	Max	Mean	Standard deviation
Input	pH	1.00	5.00	3.12	1.15
	Extractant concentration (M)	0.05	0.50	0.22	0.16
	Nitrate ion concentration (M)	0.05	1.00	0.52	0.27
	Contact time (min)	2.50	30.00	18.08	0.73
Output	Extraction efficiency (%)	6.35	95.21	50.37	0.24

### Hybrid neural-genetic algorithm

**Figure 1:**
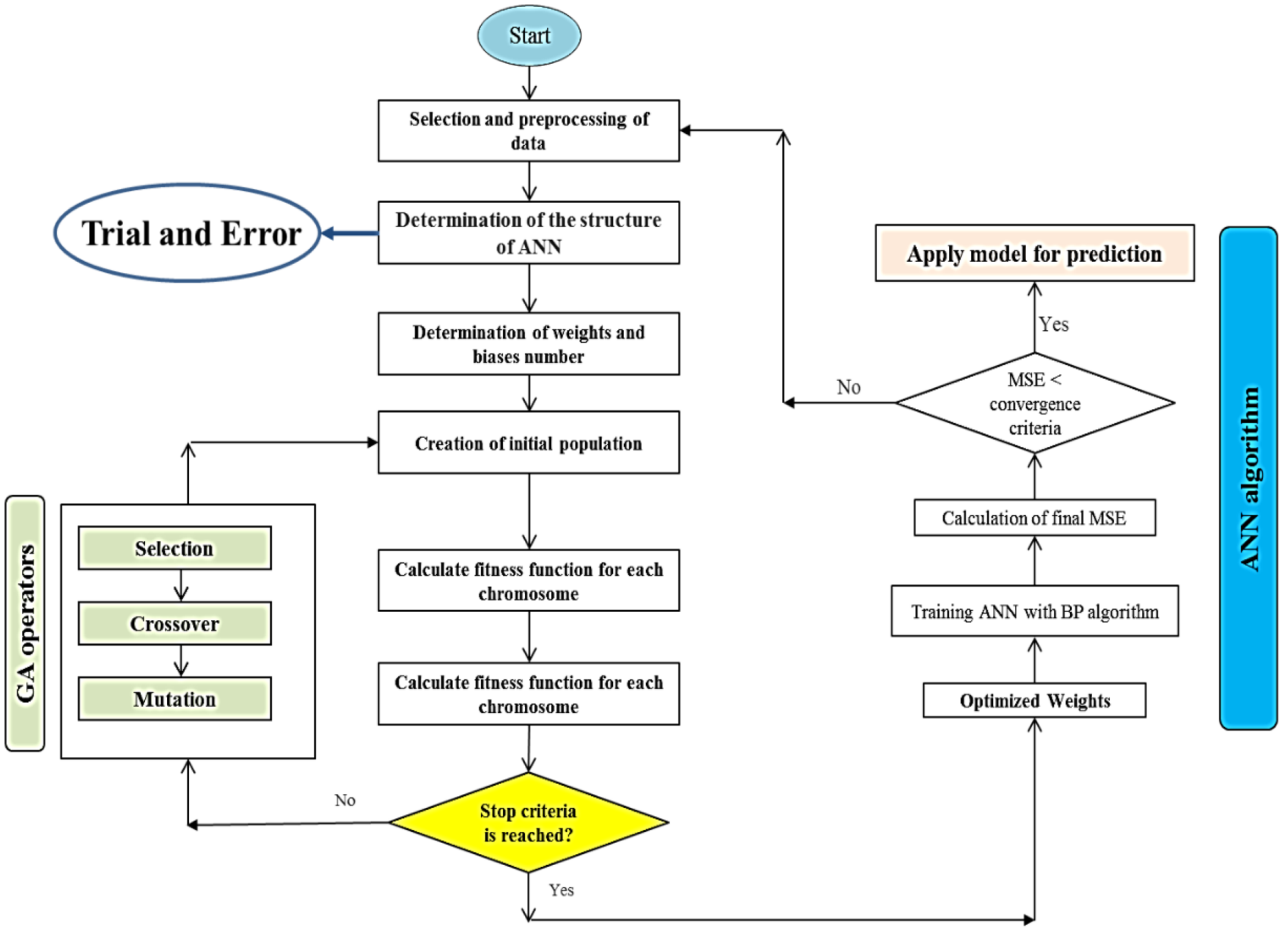
Flowchart of GA-ANN model.

The backpropagation method of training artificial neural networks acts based on the gradient descent with respect to the initial weight vector and biases to minimize the error at each iteration^45^. This method uses random initial weights and terminates based on the stopping criterion. However, this may lead to getting trapped in the local minima and slow down the convergence speed during the training phase. Therefore, to improve the efficiency of a neural network, the initial weights and the threshold of the network can be optimized using a genetic algorithm (GA)^28^. A genetic algorithm is a well-known technique for solving optimization problems^29,46^. Details about the genetic algorithm can be found in the literature^29,47^. Figure 1 shows the flowchart for the implementation strategy of GA-ANN. This methodology optimizes the search space of neural networks using a genetic algorithm, as illustrated in Fig. 1.

### Model evaluation criteria

There are different statistical criteria’s for evaluating developed models for the prediction of extraction efficiency under different operating conditions. The coefficient of determination (R^2^) and mean square error (MSE) are the most important statistical criteria, and their equations are as follows:

8 $$R^{2} = 1 - \frac{{\mathop \sum \nolimits_{i = 1}^{n} \left( {y_{i} - x_{i} } \right)^{2} }}{{\mathop \sum \nolimits_{i = 1}^{n} \left( {y_{i} - x_{i} } \right)^{2} + \mathop \sum \nolimits_{i = 1}^{n} \left( {x_{i} - \overline{x}} \right)^{2} }}$$

9 $$MSE = \frac{1}{n}\mathop \sum \limits_{i = 1}^{n} \left( {x_{i} - y_{i} } \right)^{2}$$

where n is the number of data; $\overline{x}$, x_i_, and y_i_ are the mean value of experimental data (actual data), the actual value and model output, respectively.

### Relative importance of each variable

The relative importance of each input variable on the extraction efficiency of cerium is determined using the following equation^48^:

10 $$I_{j} = \frac{{\mathop \sum \nolimits_{m = 1}^{{m = N_{h} }} \left( {\left( {\left| {W_{jm}^{ih} } \right|/\mathop \sum \nolimits_{K = 1}^{{k = N_{i} }} \left| {W_{km}^{ih} } \right|} \right) \times \left| {W_{mn}^{ho} } \right|} \right)}}{{\mathop \sum \nolimits_{K = 1}^{{k = N_{i} }} \left\{ {\mathop \sum \nolimits_{m = 1}^{{m = N_{h} }} \left( {\left( {\left| {W_{jm}^{ih} } \right|/\mathop \sum \nolimits_{K = 1}^{{k = N_{i} }} \left| {W_{km}^{ih} } \right|} \right) \times \left| {W_{mn}^{ho} } \right|} \right)} \right\}}}$$

where *W, N*, and *Ij* are connection weight, the number of neurons, and the relative importance of the j_th_ input variable on the extraction of cerium, respectively. ‘*k*’ and ‘*i*’ refer to input neurons, ‘*m*’ and ‘*h*’ refer to hidden neurons, and ‘*n*’ and ‘*o*’ refer to output neurons.

## Results and discussion

### Effect of operating parameters

**Figure 2:**
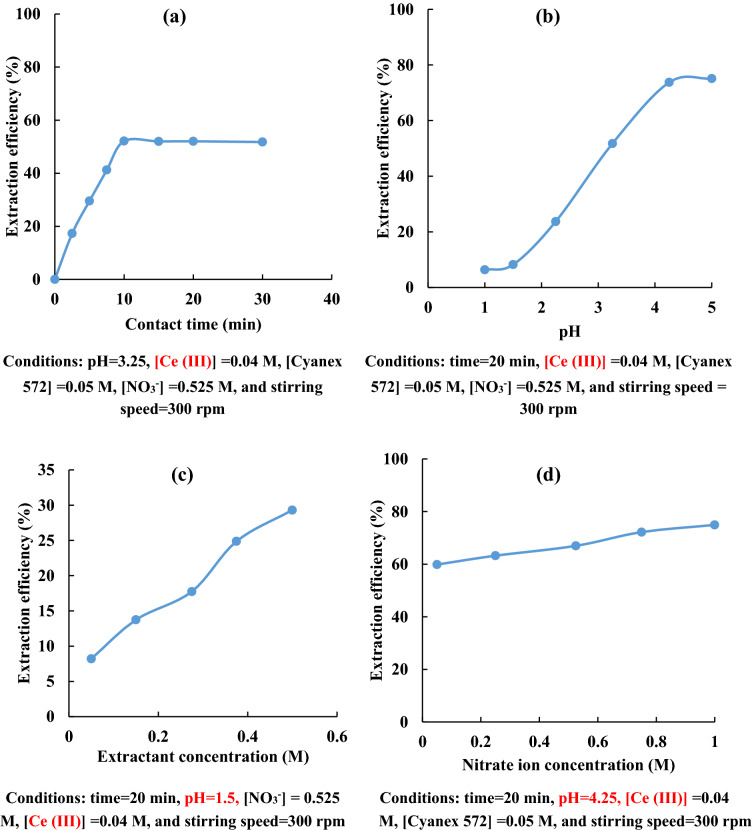
Effect of (**a**) contact time, (**b**) pH, (**c**) extractant concentration and (**d**) nitrate ion concentration on the cerium extraction efficiency.

Among various operating parameters, studies were conducted by varying the contact time and evaluating cerium extraction efficiency. The extraction of cerium versus time was shown in Fig. 2a. The amount of cerium extracted by Cyanex 572 was measured at 2.5, 5.0, 7.5, 10, 15, 20, and 30 min. The extraction efficiency at the initial time was increased drastically and reached an approximately constant value after 10 min. So, 10 min is enough time to attain equilibrium. However, a contact time of 20 min was selected to ensure completed equilibrium.

pH is one of the most important variables affecting the extraction process. The extraction of cerium by Cyanex 572 was investigated at different initial pH varying from 1.0 to 5.0. The obtained results are given in Fig. 2b. As the pH of the solution increased, the extraction efficiency of cerium from nitrate medium using Cyanex 572 was also increased. According to the Eh–pH diagram for the Ce-H_2_O system at 25 °C, the dominant species of cerium at acidic pH values (pH < 7) are Ce^3+^ and Ce(OH)^3+^. Details about the pourbaix diagram of the cerium-water can be found in literature^1^. Good extraction of cerium by Cyanex 572 by increasing pH can be attributed to the species of cerium at this range of pH (particularly pH 4–6). Similar findings were reported by Agarwal et al.^20^ while extracting cerium from nitric acid solutions by PC88A. Their results indicated that the percentage extraction of cerium was increased from 0 to 58%, with an increase in solution pH from 1.0 to 6.0.

The influence of extractant concentration on cerium extraction by Cyanex 572 was studied by varying the solvent concentration from 0.05 to 0.5 M. The obtained results are shown in Fig. 2c. As the concentration of Cyanex 572 was increased, the extraction efficiency of cerium from the nitrate medium also increased, because of the available molecules of solvent for cerium extraction increased.

The effect of nitrate ion concentration on the cerium extraction was studied by varying it from 0.05 to 1 M. The obtained results are shown in Fig. 2d. The efficiency of cerium extraction from nitrate medium increased from 59.87 to 74.93%; while increasing nitrate ion concentration from 0.05 to 1.0 M. It can be due to the salting-out effect, which is attributed to water activity changes and common ion effect caused by the addition of nitrate ion^21^.

## Extraction mechanism

**Figure 3:**
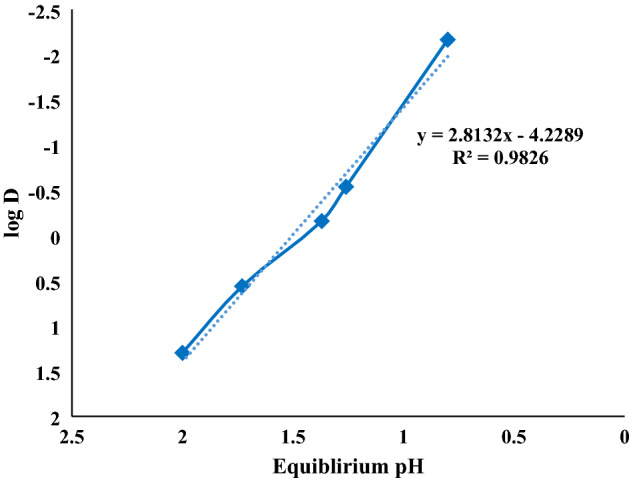
Influence of equilibrium pH on the distribution coefficient of cerium (contact time = 20 min, [Ce] = 0.04 M, [Cyanex 572] = 0.5 M, and stirring speed = 300 rpm).

Graphical analysis^41^ (slope analysis) was used to recognize the dependency between the logarithmic distribution ratio (log D) and the values of equilibrium pH (the pH of aqueous solution in an equilibrium state). As shown in Fig. 3, the plot of log D versus pH_eq_ gives a slope of 2.81, indicating that three hydrogen ions are incorporated in the chelates with cerium (III).

**Figure 4:**
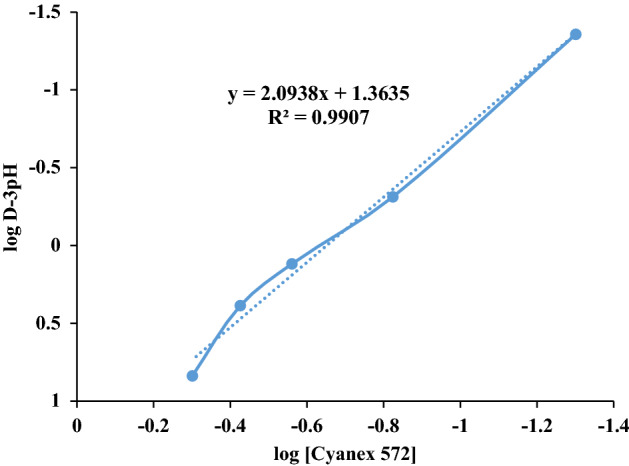
Effect of Cyanex 572 concentration on the distribution coefficient of cerium (contact time = 20 min, pH = 3, [NO_3_^−^] = 0.525 M, [Ce] = 0.04 M, and stirring speed = 300 rpm).

The influence of the Cyanex 572 concentration on the distribution ratio of cerium (III) is shown in Fig. 4. D_Ce_ increases linearly with an increase in the concentration of extractant. The plot of log D_Ce_-3pH versus log [Cyanex 572] _(org)_ gives a slope of 2.09, which indicates that the chelates of cerium (III) contain two molecules of Cyanex 572.

According to the results and above discussion, Eq. 4 could be rewritten as:

11 $$Ce^{3 + } + 2\left( {HL} \right)_{2\left( o \right)} \rightleftarrows Ce.L_{3} . HL + 3H_{\left( a \right)}^{ + }$$

Therefore, the composition of Ce (III) extracted complex by Cyanex 572 was proposed to be Ce. L_3_ HL. Also, the mean value of log K_eq_ for the above equation was found to be − 2.69.

**Figure 5:**
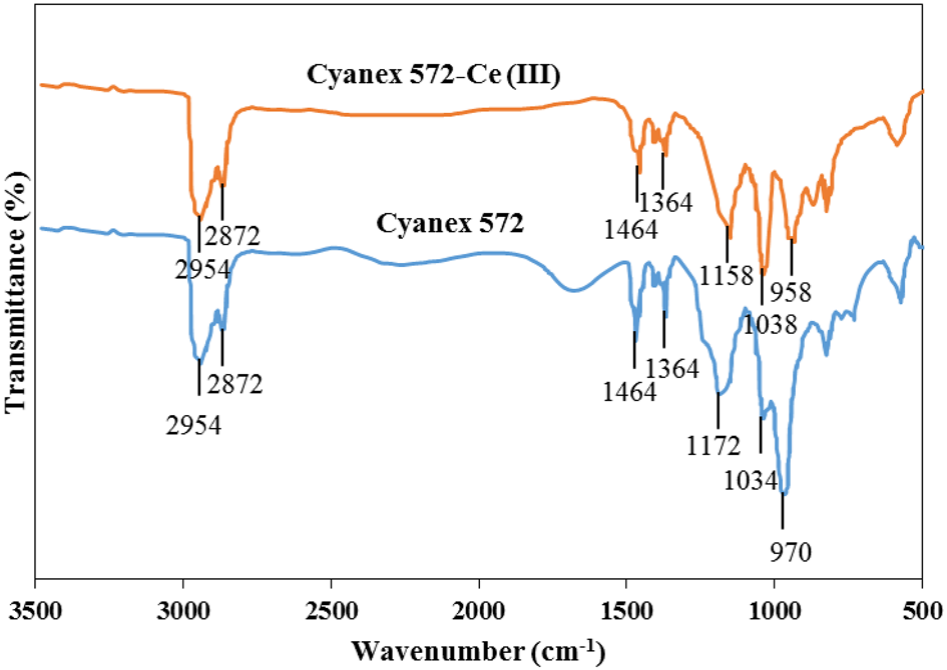
FTIR spectra of Cyanex 572 and Cyanex 572-cerium (III).

Fourier Transform Infrared Spectroscopy (FTIR) analysis needs to be conducted to determine the formed complex's structure. Figure 5 shows comparison of FTIR spectra of Cyanex 572 before and after cerium (III) loading. The vibration band belongs to the P=O bond of Cyanex 572 component appears at 1172 cm^−1^ and was shifted to 1158 cm^−1^. The observed vibration band in 970 cm^−1^ is assigned to the asymmetric stretching vibration of the P–O–C functional group, which was slightly shifted to 958 cm^−1^ after the extraction of cerium (III). These mentioned changes supported the hypothesis that cerium extraction by Cyanex 572 was performed via a cation-exchange mechanism. The IR characteristic peaks for Cyanex 572 and Cyanex 572-cerium (III) are presented in Table 3.

**Table 3:** IR characteristic peaks for Cyanex 572 and Cyanex 572-cerium (III).

Bonds	Wavenumber (cm^−1^)	
	Cyanex 572	Cyanex 572-cerium (III)
P–O–H	970	958
P–O–C	1034	1038
P=O	1172	1158
CH_3_	1364, 1464	1364, 1464
C–H	2872, 2954	2872, 2954

To compare the extraction of cerium from nitrate and nitric acid media, it is necessary to study the influence of nitric acid concentration on the extraction of nitric acid by Cyanex 572. Complex formation between nitric acid and Cyanex 572 based on the following reaction decreases the extraction of cerium.

12 $${\text{H}}^{ + } + {\text{NO}}_{3}^{ - } + {\text{L}}_{{({\text{org}})}} \leftrightarrow {\text{HNO}}_{3} \cdot {\text{L}}_{{({\text{org}})}}$$

**Figure 6:**
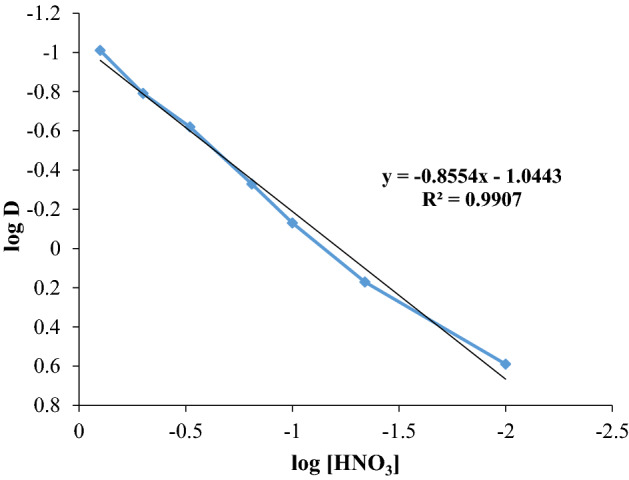
Influence of nitric acid concentration on the distribution coefficient of cerium (contact time = 20 min, [Ce] = 0.04 M, [Cyanex 572] = 0.5 M, and stirring speed = 300 rpm).

It can be found that Cyanex 572 extractant creates a complex with nitric acid and therefore, there is not enough molecules of solvent for cerium extraction. It means that the extraction efficiency of cerium decreases. On the other hand, the consumption of hydrogen ions through co-extraction of HNO_3_ and cerium decreases the extraction efficiency of cerium, as illustrated in Fig. 6. Similar results were obtained in similar solvent extraction systems^21^.

### Development of a predictive model for cerium extraction

#### Development of ANN model

In this research, input parameters to the ANN model were contact time, pH, extractant concentration, and nitrate ion concentration. The extraction efficiency of cerium was considered the output of this technique. The ANN model, consists of three inputs, hidden, and output layers, are constructed. The neural network correlates the input and the output parameters based on gradient descent to minimize the error between the actual and model output values^49^. Applied transfer functions in hidden and output layers were sigmoid and linear transfer functions, respectively^50^. To train the network, the Levenberg–Marquardt back-propagation algorithm was used^51^. To train the developed model, 70% of data (28) were randomly selected, and the rest 30% (11) were selected for testing the model. All the 39 datasets were normalized in the range of − 1 and + 1 before modeling using the following equation:

13 $$X_{Normal} = 2\frac{{X - X_{min} }}{{X_{max} - X_{min} }} - 1$$

where X_Normal_, X, X_max_, and X_min_ are normalized, original, maximum, and minimum values of the parameters, respectively. The optimal ANN structure can be determined by changing the number of hidden layers and the number of neurons in those layers (trial and error method)^52^. For this purpose, different topologies varying the number of neurons from 2 to 20 were constructed. According to the statistical observations, the structure of 4:7:1 with Levenberg–Marquardt backpropagation was selected as the best arrangement for the network.

#### Development of the GA-ANN model

**Figure 7:**
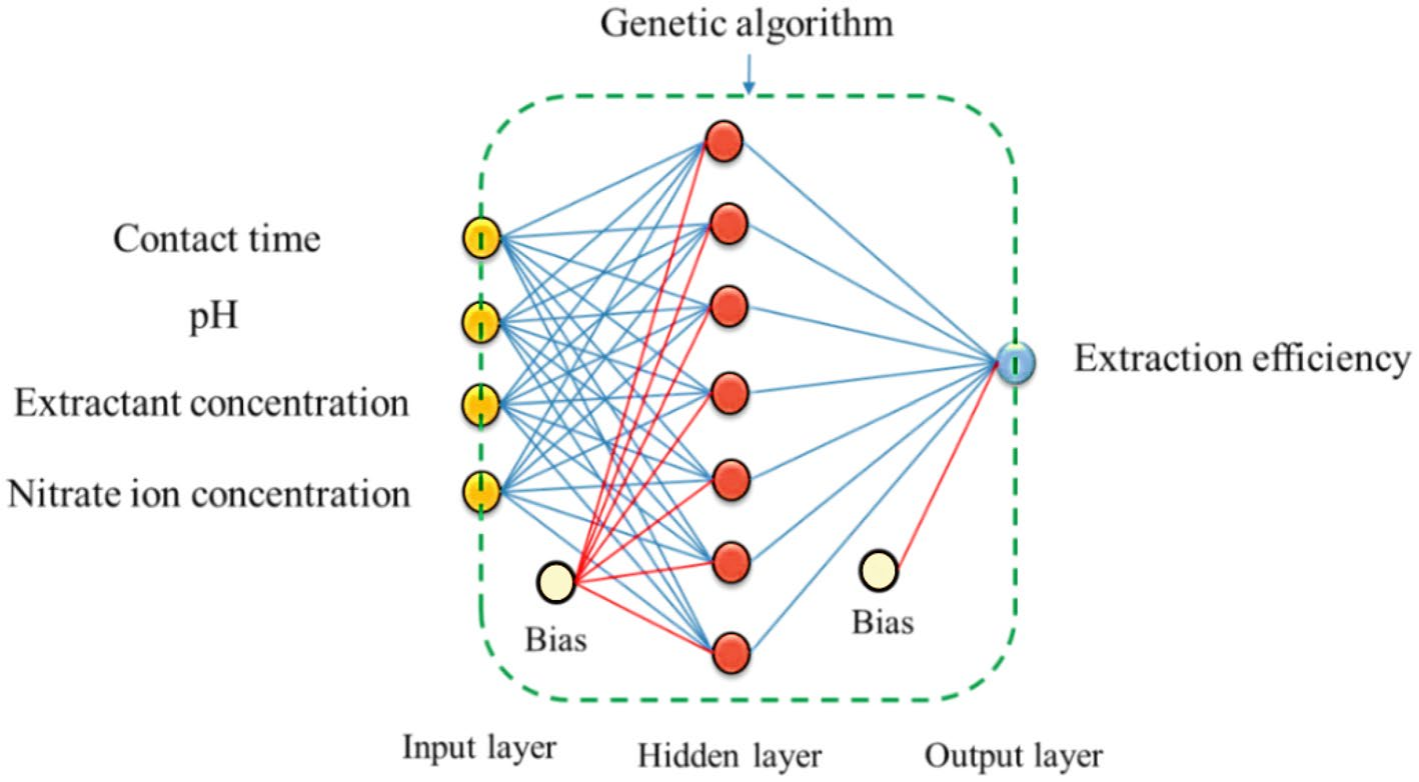
Architecture of GA-ANN implementation topology.

In the ANN models, weights were first selected randomly. These random initial weights may be resulted in trapping into the local minima and slow convergence speed in the training phase^53^. Therefore, a genetic algorithm (GA) was combined with a neural network to optimize the initial weight vector and the threshold of the network. The operators of GA include selection, crossover, and mutation. This research, used the roulette wheel selection algorithm (selection operator) was used to select individuals with larger fitness as parents for the next generation^54^. The number of generations and population size for the present study were considered 150 and 150, respectively. The multi-point crossover with the probability of 0.5 was applied to generate a new population with high diversity. The mutation operator with the probability of 0.04 was considered for altering the values of few genes of the chromosome to generate new individuals. The stopping criterion for this research was the maximum number of generations. Figure 7 shows the architecture of GA-ANN implementation topology for the present work.

**Figure 8:**
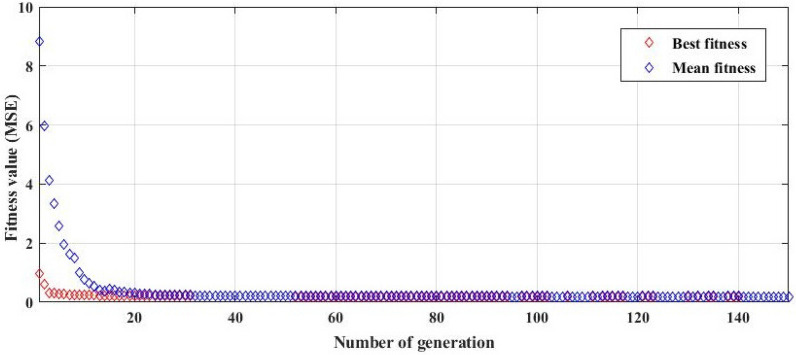
Convergence curves of the average fitness and the best fitness of evolution generation for optimizing the initial weights and biases of the network.

To improve the performance of ANN, while learning the network, the initial weights and the threshold of the network were optimized by the GA technique. The convergence curves of the average fitness and the best fitness of evolution generation for initial weight and bias are shown in Fig. 8. It can be seen that the value of MSE was decreased by increasing the number of generations. Finally, the best fitness value for the developed model was obtained at 0.1140. The connection weights and biases related to the chromosome that has the least value of MSE were considered for training the network.

**Figure 9:**
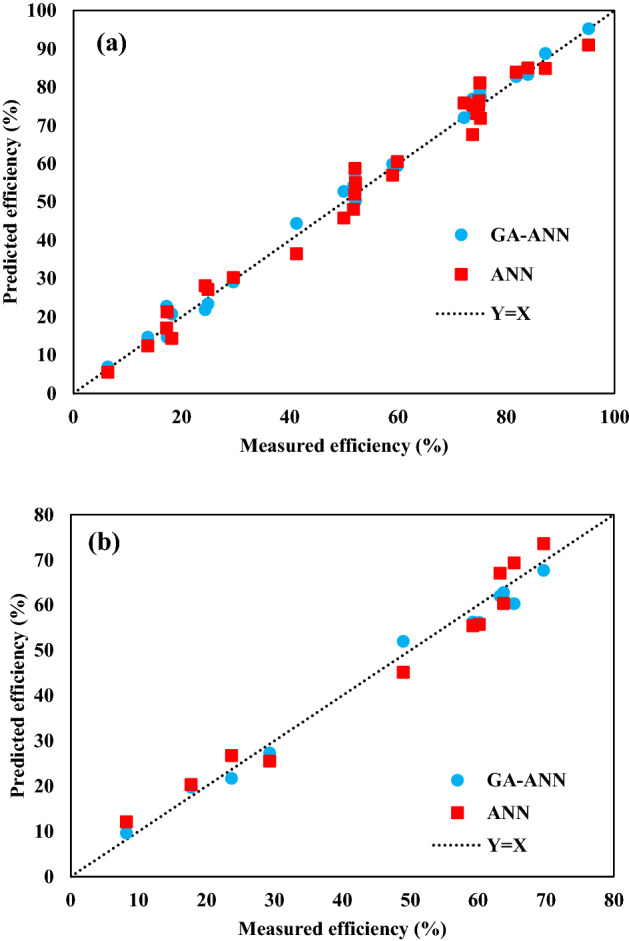
Correlation between predicted cerium extraction using ANN and GA-ANN methods vs. the measured cerium extraction for (**a**) training and (**b**) testing datasets.

After optimizing the initial weights and the threshold of the network, they are considered for back propagation training. Figure 9a,b show the correlation between predicted cerium extraction using the GA-ANN method and the measured cerium extraction for training and testing datasets, respectively. The MSE and R^2^ were obtained at 1.728 and 0.9938 for the training phase and 7.225 and 0.9899 for the testing phase, respectively. Whereas, the MSE and R^2^ for ANN approach were 10.687 and 0.9836 for the training phase and 13.827 and 0.9693 for the testing phase, respectively. Results indicated that the GA-ANN model has a higher capability than the ANN model to develop a complex nonlinear expression to predict cerium extraction by Cyanex 572.

**Figure 10:**
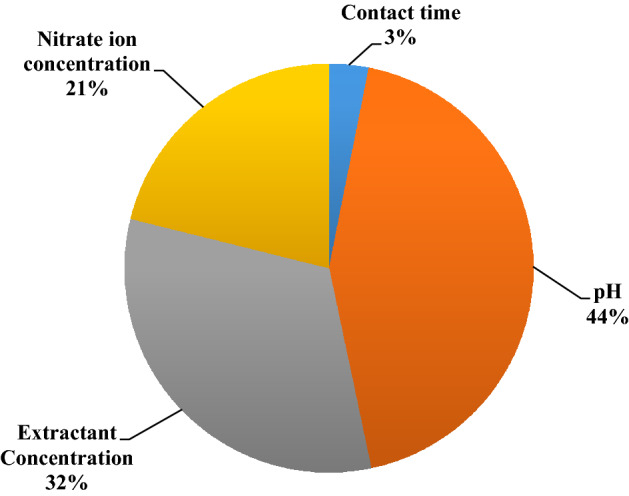
Relative importance of variables on extraction efficiency of cerium.

After optimizing the neural weight data, they were applied to calculate the relative importance of each input variable on cerium extraction. The obtained results are presented in Fig. 10. The results indicate that pH had the highest effect (44%) on cerium extraction among the studied parameters. After that, extractant concentration (32%) considerably influenced the cerium extraction, followed by nitrate ion concentration (21%) and contact time (3%). The obtained results were consistent with Table 1.

### Separation studies from other rare earth elements

Due to lanthanide contraction, the change in the atomic or ionic radii of rare earth elements is very small. Hence, their physical and chemical properties are similar^11^. Given their similar physicochemical properties, it is challenging to separate cerium from other rare earth elements^55^. The solvent extraction of Ce (III) from the aqueous solution was successfully performed using Cyanex 572^56^. But, there is no systematic report about the separation of Ce (III) from other rare earths by Cyanex 572. As presented in Table 4, the distribution coefficients of rare earths increase with an increase in the pH of a solution. The separation between cerium and neodymium seemed not be feasible because of the low value of separation factor (β = 5.8). The low values of separation factors showed the difficulties of separation for the elements of cerium and lanthanum. It is noted that the concentrations rare earths in the leach liquor solution are different from each other ([Ce] = 0.04 M, [Nd] = 0.015 M, [Pr] = 0.004 M, [La] = 0.014 M, and [Y] = 0.008 M). In addition, Pr (III) and Y (III) exhibit similar extraction behaviors when the aqueous pH values were adjusted. However, the extraction efficiency of Ce (III) is still significantly higher than that of other rare earths at higher pH values. Also, under similar conditions, the separation factors of cerium from other rare earths were improved with an increase in solution acidity. The optimum value of pH for the separation of cerium from other rare earths was found to be 3.5.

**Table 4:** Distribution coefficients and separation factors of cerium and other rare earths.

Initial pH	Distribution coefficient					Separation factor			
	D_Ce_	D_Nd_	D_La_	D_Pr_	D_Y_	β_Ce/Nd_	β_Ce/La_	β_Ce/Pr_	β_Ce/Y_
1.5	1.1	0.82	0.92	0.33	0.39	1.341	1.196	3.333	2.821
2.0	7.78	1.37	0.99	0.41	0.45	5.679	7.859	18.976	17.289
2.5	13.24	2.89	1.23	0.71	0.69	4.581	10.764	18.648	19.188
3.0	21.45	4.234	1.65	0.98	0.99	5.066	13.000	21.888	21.667
3.5	44.23	7.76	1.9	1.21	1.22	5.700	23.279	36.554	36.254
4.0	62.45	14.12	2.35	1.78	1.79	4.423	26.574	35.084	34.888

Significant values are in underline.

## Conclusions

A systematic study of the solvent extraction of Ce (III) was carried out using Cyanex 572 diluted in kerosene from nitrate media. The effects of contact time, pH, extractant concentration, and nitrate ion concentration were investigated to characterize the Cerium extraction behavior. Based on these results, an extraction mechanism was suggested. By specific extraction experiments and FTIR spectra analysis, a cation exchange mechanism was proposed, and the stoichiometry of Cyanex 572 to Ce (III) was found to be 2:1. Then, these operating parameters of cerium extraction were considered input for model development based on intelligent techniques (ANN and GA-ANN). Results indicated that the GA-ANN model has a higher capability than the ANN model to understand the mechanisms and predict cerium extraction by Cyanex 572. By optimizing the initial weights and biases, the GA-ANN model outperforms the ANN model significantly. The relative importance of each studied variable on cerium extraction was also evaluated using the neural weight data. Results indicated that pH is the most effective parameter for cerium extraction (44%), followed by extractant concentration (32%), nitrate ion concentration (21%), and contact time (3%). Finally, the separation of Ce (III) from other rare earths like La (III), Nd (III), Pr (III), and Y (III) was investigated using this extraction system. The results illustrated that the present system provides better separation of cerium from heavy rare earths than light rare earths. Overall, it can be concluded that Cyanex 572 would be an effective extractant for the separation and enrichment of Cerium from various resources.
